# Politics matters for individual attitudes toward vaccine donation: cross-national evidence from the United States and Taiwan

**DOI:** 10.1186/s12992-023-00940-x

**Published:** 2023-06-20

**Authors:** Yuan Hsiao, Fang-Yu Lin, Greg Chih-Hsin Sheen, Ching-Hsing Wang

**Affiliations:** 1 Department of Communication, University of Washington, Communications Building, Seattle, WA 98195-3340 USA; 2 Department of Public Health, National Cheng Kung University, No.1, University Road, Tainan City, 701 Taiwan; 3 Department of Political Science, National Cheng Kung University, No.1, University Road, Tainan City, 701 Taiwan

**Keywords:** COVID-19, Vaccines, Vaccine donations, Conjoint experiment, Diplomatic relations

## Abstract

**Background:**

Vaccine equity has been a major concern during the COVID-19 pandemic. According to the principle of vaccine equity, donor countries should apply the criterion of needs to make decisions about vaccine donation instead of considering recipient countries’ economic status. We examine whether people follow the same criterion or consider other factors to decide which country to donate vaccines and how many vaccines should be delivered.

**Methods:**

We conducted online surveys with the design of conjoint experiment in the United States and Taiwan in 2021. 1,532 American citizens and 1,587 Taiwanese citizens were interviewed. The respondents were broadly quota-matched to their respective demographic proportions on the dimensions of age, gender, and education. We estimated the average marginal component effects (AMCEs) of the conjoint attributes by using the OLS regression models with standard errors clustered at the respondent level.

**Results:**

15,320 and 15,870 decisions on vaccine donation generated by conjoint experiment respectively in the United States and Taiwan were included in the analysis. Both American and Taiwanese people tend to donate vaccines to countries that suffer severe consequences of COVID-19 and democracies compared to authoritarian countries. However, they are less willing to donate vaccines to those with higher levels of capability in response to COVID-19. Taiwanese people tend to donate vaccines to countries having formal diplomatic relations with Taiwan (AMCE 13.4%, 95% CI 11.8%-15.1%). Nonetheless, American people would rather donate vaccines to countries without formal diplomatic relations with the United States (AMCE − 4.0%, 95% CI -5.6%--2.4%).

**Conclusions:**

The findings reveal that politics plays a significant role in people’s decisions about vaccine donation. Under electoral pressure, political leaders must think about how to respond to the public’s preferences over vaccine donation to achieve vaccine equity and address the global health crisis.

**Supplementary Information:**

The online version contains supplementary material available at 10.1186/s12992-023-00940-x.

## Introduction

It has been more than three years since the World Health Organization (WHO) declared COVID-19 a pandemic, and many countries have still struggled to manage a continuing COVID disease burden. According to the latest available data from the WHO in May 2023, more than 765 million people have been infected with COVID-19 and more than 6.92 million people have died from COVID-related causes worldwide. In addition to the tragic losses of life and health, the COVID pandemic has caused massive economic and social disruption [1]. To fight against COVID-19, vaccines has been well-recognized as an effective tool to contribute to the control of the pandemic and could provide substantial protection against severe disease and death [2].

Although more than 5.5 billion people worldwide have received at least one dose of COVID-19 vaccine, equal to about 69% of the world population in May 2023, there exists a stark gap between vaccination programs across countries. While the WHO and the COVID-19 Vaccines Global Access (COVAX), a vaccine-sharing scheme founded in April 2020, have strived to ensure fair and equitable access to COVID-19 vaccines, inequality in the distribution of COVID-19 vaccines across countries remains. Wealthy countries have higher COVID-19 vaccination rates compared to poor nations [3]. Due to unequal access to COVID-19 vaccines across countries, UN Human Rights has strongly proclaimed thatCOVID-19 vaccines should be treated as global public goods and affordable to all and accessible without discrimination around the world. Health is a human right that must be protected and thus all countries and pharmaceutical companies should work together to ensure equitable, affordable, timely and universal access to vaccines in response to the COVID-19 pandemic [4]. More than one billion doses of COVID-19 vaccine have been shipped via the WHO and COVAX to primarily low- and middle-income countries. Despite the fact that the COVAX initiative presents an effective means to hasten the global distribution of vaccines, the efforts of donor nations and industries to pursue their own interests in areas such as national security, diplomacy, and commerce have weakened the role of COVAX in promoting equitable access to COVID-19 vaccines worldwide. Therefore, it is necessary to allocate vaccines to all countries based on their needs, without regard for their economic status, in order to achieve global vaccine equity [5].

Besides, some countries have donated COVID-19 vaccines to those in need. For instance, China has donated 119 million vaccine doses to dozens of countries, whereas the United States has donated 275 million vaccine doses to over 110 countries. Besides, the United States has pledged to donate at least 1.1 billion doses of COVID-19 vaccine for global use before 2023 and Germany has planned to donate 75 million vaccine doses to low- and middle-income countries in 2022 [6]. Given that affordable, non-discriminatory access to the vaccine is a human right, donor countries should treat those countries in need of COVID-19 vaccines equally. Moreover, since vaccines have been regarded as the most effective approach to preventing serious illness during the COVID-19 pandemic, it is required to take the issue of vaccine inequality more seriously. Nonetheless, the ongoing inequality in vaccine distribution has remained one of the greatest failures of international cooperation to combat COVID-19.

Instead of asking how political leaders in donor countries decide which countries should be given COVID-19 vaccines, this study examines what factors influence the decision on vaccine donation from the public’s perspective. It is of significance to investigate how people view vaccine donation because they want political leaders to effectively spend their tax dollars on foreign aid. Especially given the severity of the domestic COVID-19 situation, political leaders must justify their decisions on vaccine donation to meet public expectations. Once political leaders could not provide justification for vaccine donation, they might get blamed and pay the price in the next election. Therefore, there is a pressing need to understand what factors drive the public’s preferences over vaccine donation.

A growing body of literature has assessed the principles to guide vaccine donation [7], the importance of equitable access to vaccination [8], and public support for vaccine donation [9] during the COVID-19 pandemic. Although certain studies have found that individuals generally favor donating COVID-19 vaccines to low- and middle-income countries [10, 11], there is limited knowledge about what other factors may be influencing individual preferences regarding vaccine donations. Built on conceptions of empathy [12], distributive justice [13], and international relations [14], we develop fairness-based and political explanations of public preferences over vaccine donation. Specifically, this study examines how individuals’ decisions regarding vaccine donation are influenced by their perceptions of recipient countries’ COVID-19-related suffering, response capacity, and political situation. First of all, when theorizing about how governments allocate foreign aid, there are two main arguments that center around either the interests of donor countries or the needs of recipient countries. The former contends that donor countries use aid to promote their national interests [15], whereas the latter emphasizes the humanitarian perspective that foreign aid serves as an altruistic policy instrument to alleviate suffering in the recipient countries [16]. While donor countries might obtain some benefits by donating vaccines, this study argues that as for vaccine donation, more emphasis should be placed on humanitarian considerations given that the COVID-19 pandemic is the greatest public health crisis that the world has faced in the past century. The WHO has also urged developed countries to share vaccines with countries in need for the sake of global public health. Countries with high numbers of COVID-19 cases and deaths are likely experiencing significant humanitarian challenges. Donating vaccines to these countries can help to provide much-needed relief to affected populations and prevent the spread of the virus across borders. Previous research has demonstrated that donor countries prefer to donate vaccines to countries with higher burden of COVID-19 [17]. Accordingly, it is inferred that people should be more likely to donate vaccines to countries that are experiencing high levels of COVID-19 cases and deaths. In other words, if people are empathetic about the COVID-19 situation in a country, we expect that the greater number of COVID-19 cases and deaths experienced by that country would correspond with an increased willingness among individuals to donate vaccines.

Second, individual preferences over vaccine donation may also be explained by countries’ competencies based on the ability-to-pay principle. Although the ability-to-pay principle is a taxation concept that the amount of taxes people pay should be decided by the amount they earn [18], this idea could be extended to understand how people make decisions on vaccine donation. Given distinct country differences in affluence and vaccine development, some countries are more capable of delivering vaccines to their people than other countries. Therefore, individual decisions on vaccine donation should mirror differences in wealth among countries. One study conducted in the United States indicates that individuals are more inclined to donate vaccines to low- and middle-income countries [10]. Similarly, a study from the United Kingdom finds that there is strong public support for COVID-19 vaccine donations to low-income countries [11]. Furthermore, another study using country-level data shows that countries which donate vaccines tend to select countries with a lower GDP per capita. These results suggest that wealthy countries possess the capabilities to obtain sufficient vaccines for their own populations and may not require aid in this regard. By contrast, poor countries are in more urgent need of COVID-19 vaccines due to lack of resources to purchasing them [19]. That is, from the perspective of distributive justice, people will feel a moral obligation to assist impoverished countries in obtaining vaccines since these countries lack the resources to secure an adequate supply of vaccines for their people. Consequently, we expect that individuals will exhibit a preference for donating a greater number of vaccines to countries that are economically disadvantaged.

Finally, people may also regard vaccine donation from a political perspective that highlights the role of diplomatic relationship and regime type. Institutional arrangements play a critical role in fostering interaction between countries. Countries which have diplomatic relations with each other should have better cooperation and be more willing to offer help when their allies are in trouble. Studies demonstrate that diplomatic relationships could boost trade [20], military cooperation [21], and development aid between countries [22]. In addition, a study has found that donor countries prefer to donate vaccines to those countries with closer trade relations [17]. Therefore, it makes sense to extend the importance of interstate or diplomatic ties to elaborate individual decisions on vaccine donation. That is, if individuals are aware that countries with established diplomatic ties to their own nation require assistance, they are more likely to feel a sense of obligation to aid their allies instead of countries without formal relations, or even adversaries. Therefore, we anticipate that people will be more inclined to donate vaccines to countries that have established diplomatic relations with their own.

On the other hand, the argument of regime type highlights that people have different degrees of favorability for democratic and authoritarian countries. A Pew Research Center survey conducted in 38 countries in 2017 reveals that there is global support for representative and direct democracy [23]. Regime type could influence foreign policy behavior that democracies tend to behave more cooperatively and less conflictingly in the international system especially when they interact with other democracies. However, authoritarian countries tend to treat democracies with more conflictual and less cooperative behavior [24]. Regime type also affects interstate cooperation on commercial issues. As countries become more democratic, they are more likely to conclude trade agreements [25]. Therefore, people should hold more favorable attitudes toward democracies than authoritarian countries. Given that vaccines should be available to everyone in the world regardless of what type of regime their countries is, there is empirical merit to examine whether people would prefer to give more vaccines to democracies than authoritarian. Ideally, there should be no discrimination between democratic and authoritarian countries when it comes to vaccine donation. However, due to significant differences in public preferences toward democratic and authoritarian regimes, it is expected that individuals are more willing to donate vaccines to democracies than authoritarian countries. We anticipate that the findings will reveal whether individuals hold divergent inclinations regarding vaccine donation to democratic versus authoritarian countries.

In the light of the above discussion, we argue that three types of factors – damage due to COVID-19, national competency, and political consideration – would drive individual decisions on vaccine donation. Specifically, we hypothesize that people are more likely to donate vaccines to countries that have encountered more pronounced COVID-19 challenges, have limited resources or capacity to deal with the pandemic, are democratic in nature, and share diplomatic relationships with their own countries. To assess the impact of the above-mentioned factors on individual preferences over vaccine donation, we conduct a cross-national study by fielding a survey experiment in the United States and Taiwan. We select United States and Taiwan to be included in the current analysis mainly because these two countries have developed their COVID-19 vaccines and donated vaccines to other countries. The difference between these two countries lies in the fact that the United States is the world’s largest donor of COVID-19 vaccines, whereas Taiwan has not only donated vaccines but also received vaccines donated by some countries. While both the United States and Taiwan are democracies, they have different international statuses. The United States is a global power and has formal diplomatic relations with most countries in the world. However, Taiwan’s international legal status has been an issue because China has claimed Taiwan to be part of its territory. Under China’s foreign interventions, Taiwan only has full diplomatic relations with 13 countries as well as Vatican City at present. Therefore, the analyses of the United States and Taiwan would provide new insights into the role of politics in individual attitudes toward vaccine donation.

## Methods

### Participants

We conducted cross-national online surveys with the design of conjoint experiment in the United States and Taiwan in November and December 2021. Ethical approval was given by the Institutional Review Board at National Cheng Kung University (approval number is NCKU HREC-E-110-522-2) for both online surveys. All participants provided informed consent at the start of the survey, and no forms of deception or hidden purpose existed, so all aspects were fully explained. We used Qualtrics to create online surveys and entrusted Rakuten Insight to respectively interview 1,532 American citizens and 1,587 Taiwanese citizens, who were broadly quota-matched to their respective demographic proportions on the dimensions of age, gender, and education. In the survey of the United States, the dimension of ethnicity is also used for quota-matching. Table 1 presents information about sample distributions and national demographic proportions in the United States and Taiwan). The demographic distributions of our samples in the United States and Taiwan are not quite identical to target populations. While the proportions of female respondents are slightly similar to those in the populations of the United States and Taiwan, there are obvious differences in the age distributions between our samples and target populations. Specifically, American respondents are older than the population of the United States, whereas Taiwanese respondents are younger than the population of Taiwan. In terms of education, American respondents’ educational levels are about the same as those in the population of the United States, but Taiwanese respondents’ educational levels are higher than those in the population of Taiwan. Besides, the ethnic distribution of respondents is similar to that of the United States, although there are fewer Hispanic respondents and more respondents of other races.

**Table 1:** Comparison between demographic distributions of survey respondents and populations

	United States		Taiwan	
	Sample	Population	Sample	Population
Gender (%)				
Female	49.7%	51.6%	49.7%	51.0%
Age (%)				
18–29 (20–29)	12.1%	21.0%	26.7%	15.9%
30–39	20.7%	15.0%	27.2%	17.9%
40–49	18.1%	14.7%	27.8%	19.4%
50–59	19.8%	16.2%	13.8%	18.7%
60 or older	29.3%	33.1%	4.5%	28.1%
Education (%)				
College degree or above	33.9%	34.6%	62.1%	36.9%
Ethnicity (%)				
White	68.9%	66.7%		
Black	11.1%	12.8%		
Hispanics	4.1%	12.9%		
Other	15.9%	7.6%		

### Procedures

The potential survey participants received email invitations to participate in our online surveys. If the respondents consented to take part in the study after reading the research-related information, they clicked on the link in the email to answer the questions. In the beginning of our conjoint experiment, the respondents were presented with the following statement:

Given the continual spread of COVID-19 around the world, many countries have an urgent need for vaccines. However, the global shortage and uneven distribution of vaccines have caused significant differences in the number of vaccines available across countries. In each of the following pages, you will be shown a table with two country profiles. For each pair, read each profile carefully and indicate how you want to distribute COVID-19 vaccines.

Then the respondents were presented with a series of tables showing profiles of two hypothetical countries with different attribute values along with our two outcome questions related to which country to donate vaccines and what percentage of vaccines should be delivered (please see the appendix regarding the instructions for our conjoint experiment). The conjoint experiment contained six dimensions tied to the theoretical expectations: COVID-19 cases, COVID-19 deaths, gross national income per capita, fully vaccinated rate, formal diplomatic relation, and political system. For each dimension, we used between two and three different values.

First of all, the numbers of COVID-19 cases and deaths were used to examine the role of damages due to COVID-19, namely recipient country’s needs. The number of COVID-19 cases included three values (50,000 people, 150,000 people, 300,000 people), whereas the number of COVID-19 deaths had two values (1,000 people, 4,000 people). Second, gross national income per capita and fully vaccinated rate were used to gauge countries’ capabilities to manage COVID-19. The former included three values (US$2,500, US$8,500, US$13,000 in the United States; NT$80,000, NT$240,000, NT$360,000 in Taiwan), whereas the latter also had three values (10%, 25%, 40%). Finally, formal diplomatic relation and political system were used to operationalize political consideration. Each of them had two categories. Formal diplomatic relation indicated whether the country had formal diplomatic relation with the respondent’s country (Yes, No) and political system illustrated whether the country is a democratic or authoritarian country (democracy, authoritarianism). The number of attributes in this study generated 216 unique country profiles (3 × 2 × 3 × 3 × 2 × 2). While some might concern that survey satisfaction and respondent fatigue would pose a threat to measurement validity, previous research has documented that the quality of the subjects’ responses does not diminish even after completing 30 conjoint tasks [26].

The conjoint experiment fully randomized all attribute values. After seeing two countries with different attribute values, each respondent was asked to indicate to which country s/he wanted to donate vaccines and the percentage of vaccines s/he wanted this country to receive. Each respondent was asked to complete this task five times, meaning that they evaluated a total of 10 hypothetical countries. Consequently, we had 15,320 and 15,870 decisions on vaccine donation respectively in the United States and Taiwan.

### Statistical analysis

After data collection, we converted the raw data into a structure that is suitable for conjoint analysis. We used Stata, version 17.0 with the package of “conjoint” to perform conjoint analysis. We estimated the effects of the conjoint attributes by regressing which country to donate vaccines and the percentage of vaccines a respondent allocated to a country on treatment variables that indicate the randomly assigned attribute values for each of six attributes. By randomly assigning attribute levels, we can estimate the causal effect which is the average marginal component effect (AMCE) through ordinary least squares (OLS) regression. The OLS regression method in conjoint analysis is a straightforward and robust approach to computing various forms of respondent utilities. The OLS model’s appeal lies in its capacity to calibrate respondent preferences using rating scales instead of rankings. Additionally, the OLS method offers the significant advantage of providing standard errors for the estimated parameters. The OLS regression has become the de facto standard in conjoint analysis due to its ability to accommodate designs with numerous attributes and levels. In this study, the AMCE for a particular attribute represents the mean difference in the respondents’ binary choices between two countries differing in its levels averaged across all possible combinations. The conjoint analysis allows us to estimate how COVID-19 cases, COVID-19 deaths, gross national income per capita, fully vaccinated rate, formal diplomatic relation and political system influence individual attitudes toward vaccine donation. All *p*-values were two-sided and considered statistically significant at an *α* level of 0.05.

## Results

**Fig. 1:**
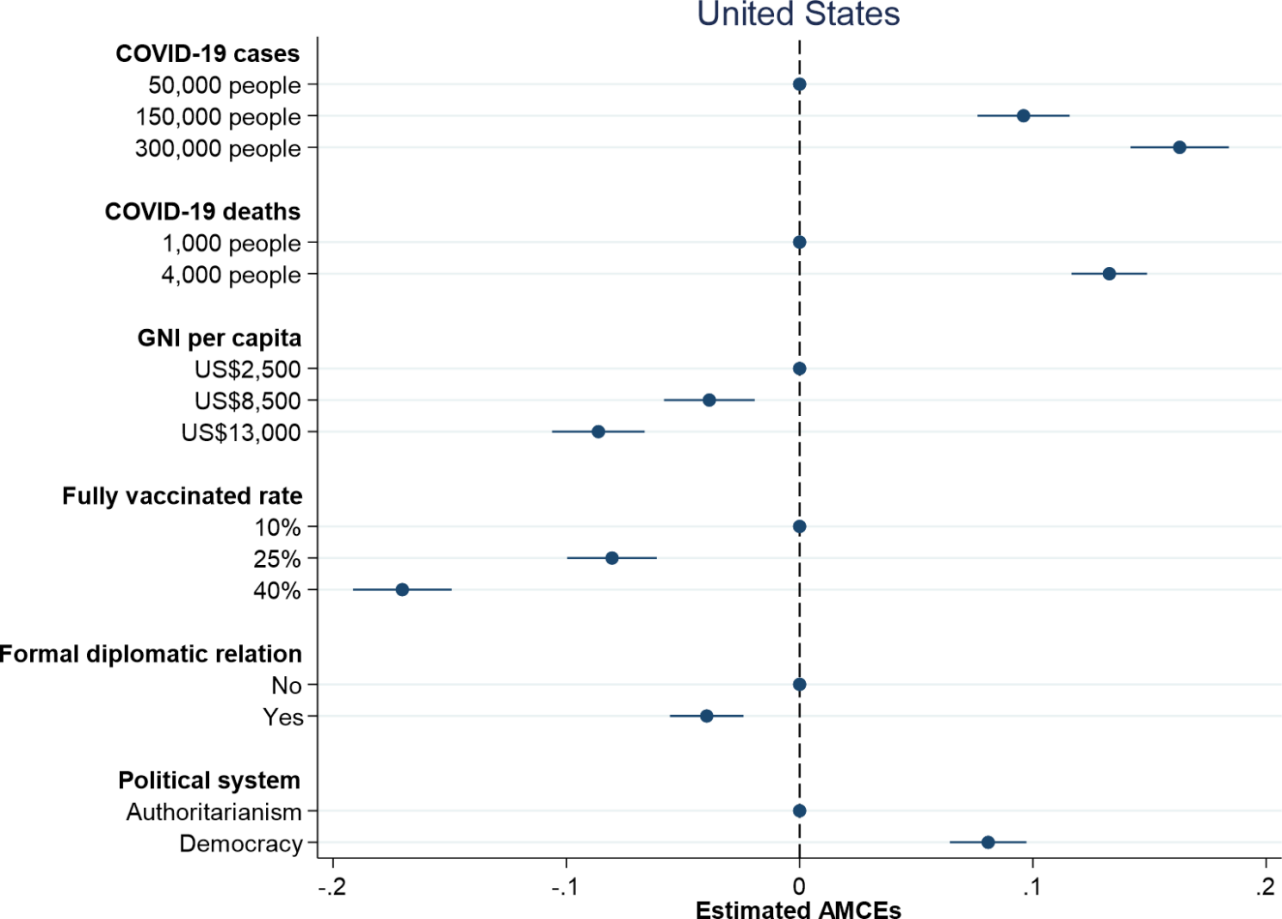
Probability of vaccine donation among American respondents

**Fig. 2:**
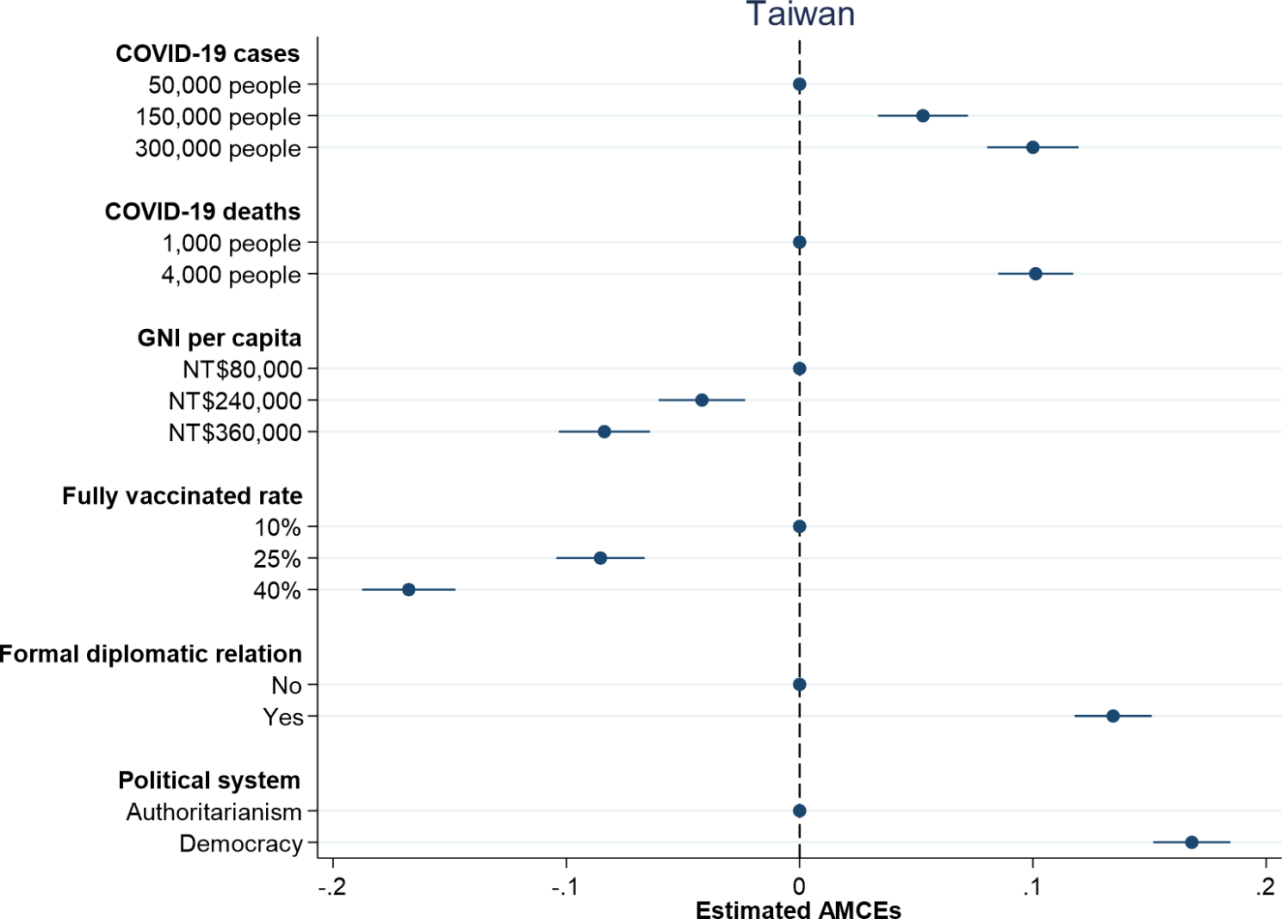
Probability of vaccine donation among Taiwanese respondents

Figures 1 and 2 respectively report the causal effects on which country to donate vaccines along with 95% confidence intervals in the United States and Taiwan. Dots without 95% confidence intervals represent the reference categories. All treatment indicators for the dimension of damages due to COVID-19 have positive and significant effects on individual preferences over vaccine donation in both the United States and Taiwan. An increase in the number of COVID-19 cases from 50,000 people to 150,000 people leads to an increase in the probability of donating vaccines by 9.6% in the United States and 5.3% in Taiwan. When the number of COVID-19 cases increases to 300,000 people, the probability of donating vaccines rises to 16.3% in the United States and 10.0% in Taiwan. On the other hand, an increase in the number of COVID-19 deaths from 1,000 people to 4,000 people causes an increase in the probability of donating vaccines by 13.3% in the United States and 10.1% in Taiwan. Overall, larger damages including COVID-19 cases and deaths trigger people to be more willing to donate vaccines, which is consistent with the argument that humanitarian considerations help explain individual preferences over vaccine donation.

When examining the impact of countries’ competencies, both proxy indicators – GNI per capita and fully vaccinated rate – exert negative and significant effects on individual preferences over vaccine donation in the United States and Taiwan. American and Taiwanese respondents are less willing to donate vaccines to countries with higher levels of GNI per capita and higher percentages of population fully vaccinated (see Figs. 1 and 2). An increase in GNI per capita from $2,500 to $13,000 generates a decrease in the probability of donating vaccines by 8.6% in the United States, whereas an increase in GNI per capita from NT$80,000 to NT$36,000 produces a decrease in the probability of donating vaccines by 8.4% in Taiwan. Besides, an increase in fully vaccinated rate from 10 to 40% leads to a decrease in the probability of donating vaccines by 17.0% in the United States and 16.8% in Taiwan. In general, the effects of countries’ competencies on individual decisions on vaccine donation in the United States are similar to those in Taiwan and the findings support the argument that people are more willing to help those countries in need.

How important are political considerations for understanding individual preferences over vaccine donation? Figures 1 and 2 display slightly different results in the United States and Taiwan. While both American and Taiwanese respondents prefer to donate vaccines to democracies compared to authoritarian countries, they think in different ways when taking formal diplomatic relation into consideration. In line with our theoretical expectation, Taiwanese respondents are more willing to donate vaccines to countries with which Taiwan has formal diplomatic relations. American respondents, however, tend to give vaccines to countries with which the United States has no formal diplomatic relations. When a country in need of COVID-19 vaccines is an ally, the probability of donating vaccines increases by 13.4% in Taiwan but decreases by 4.0% in the United States. This might reflect different international statuses of the United States and Taiwan. Given China’s aggressive diplomatic efforts to isolate Taiwan and even putting pressure on the WHO to exclude Taiwan from key meeting [27], Taiwanese people think highly of countries with formal diplomatic relations with Taiwan and are more willing to help them during the COVID-19 pandemic. By contrast, the United States is a superpower and has played a dominant role in international affairs. Since the United States has formal diplomatic relations with almost all countries, American people might tend to assist countries without official diplomatic ties with the United States out of a moral responsibility given that those countries might have difficulties in obtaining needed assistance [28]. Although the respondents in both the United States and Taiwan prefer vaccines donated to democracies, they give different weights to formal diplomatic relation in making their decisions on vaccine donation.

**Fig. 3:**
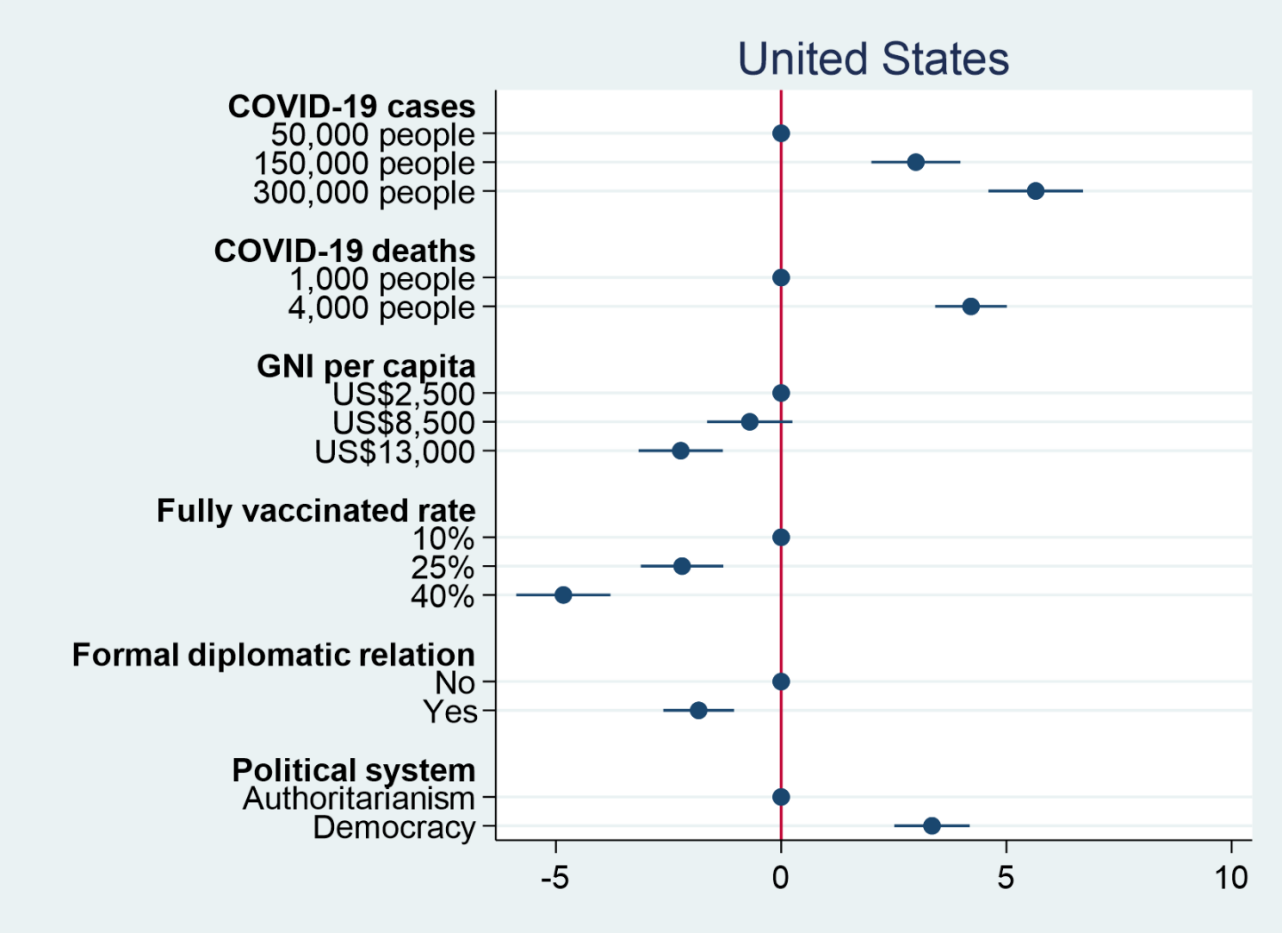
Percentage of vaccine donation among American respondents

**Fig. 4:**
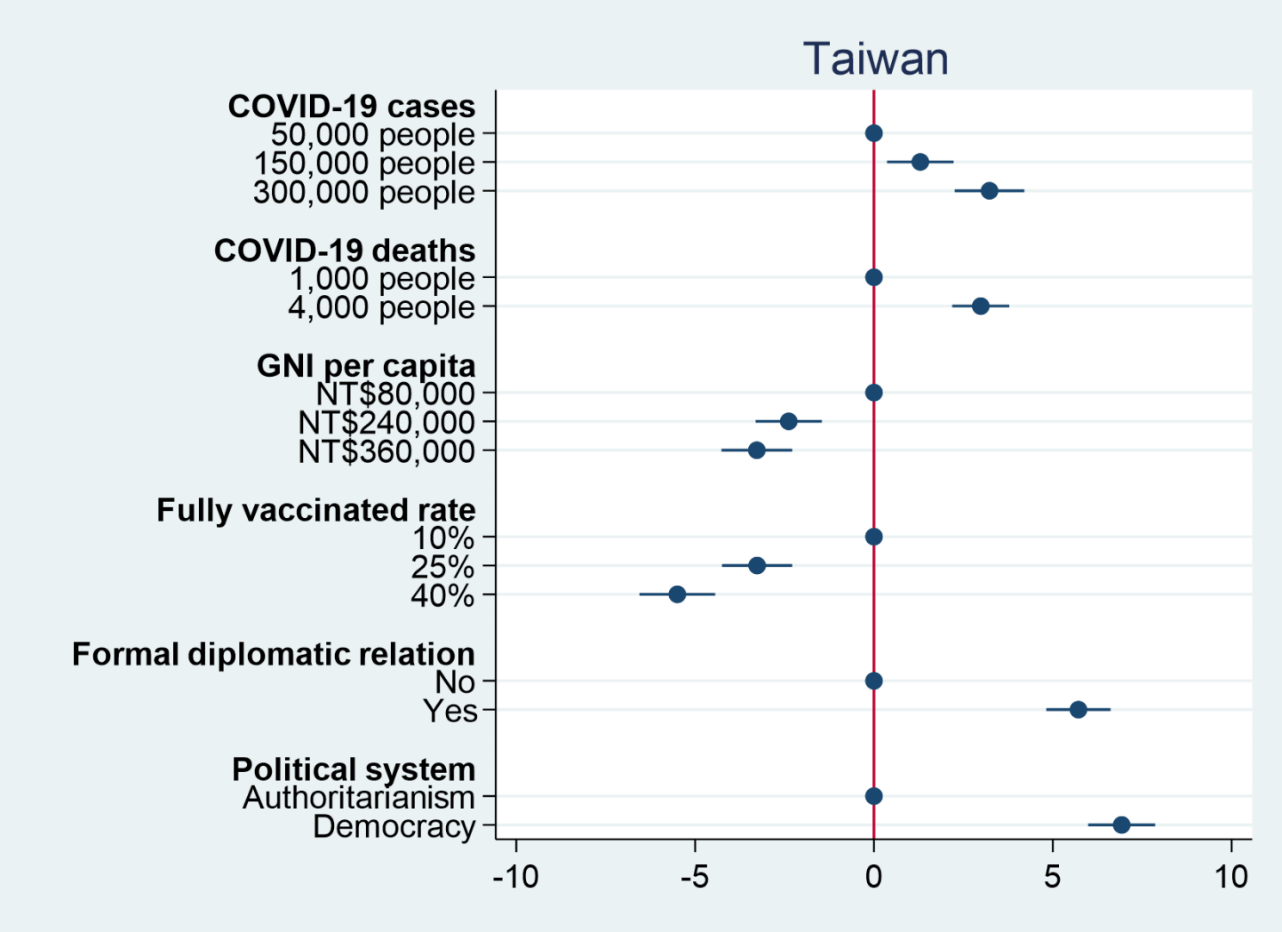
Percentage of vaccine donation among Taiwanese respondents

We then turn our attention to the percentage of vaccines donated to a country. Figures 3 and 4 respectively show the causal effects of various conjoint attributes on individual preferences over the percentage of vaccine donation in the United States and Taiwan. Similar to previous results for individual preferences over vaccine donation, the numbers of COVID-19 cases and deaths have significant positive effects on individual decisions on the percentage of vaccine donation. An increase in the number of COVID-19 cases from 50,000 people to 300,000 people causes an increase in the percentage of vaccine donation by 5.6% in the United States and 3.2% in Taiwan. When the number of COVID-19 deaths increases from 1,000 people to 4,000 people, the percentage of vaccine donation rises by 4.2% in the United States and 2.9% in Taiwan. The results again confirm the humanitarian perspective that people would distribute more vaccines to the more severely affected countries. On the other hand, both GNI per capita and fully vaccinated rate as proxies for countries’ competencies have significant negative effects on individual decisions on the percentage of vaccine donation. People would decrease the percentages of vaccine donation when a country facing COVID-19 has a higher level of GNI per capita and a higher fully vaccinated rate. This suggests that when a country has COVID-19 response capacity, people tend to think that it does not need external assistance. As for political considerations, American and Taiwanese respondents prefer to donate more COVID-19 vaccines to democracies than authoritarian countries. Nevertheless, American respondents prefer to deliver more COVID-19 vaccines to countries with which the United States has no formal diplomatic relations, but Taiwanese respondents would rather give more vaccines to Taiwan’s allies. The findings are consistent with our previous results for individual decisions on which country to donate vaccines. Overall, regardless of how outcome variables are operationalized, we consistently find that people tend to donate vaccines to countries that suffer severe consequences of COVID-19, but are less willing to donate vaccines to those with higher levels of capability in response to COVID-19. Although people generally prefer to give vaccines to democracies compared to authoritarian countries, formal diplomatic relation plays different roles in driving individual preferences over vaccine donation among people living in different countries.

**Fig. 5:**
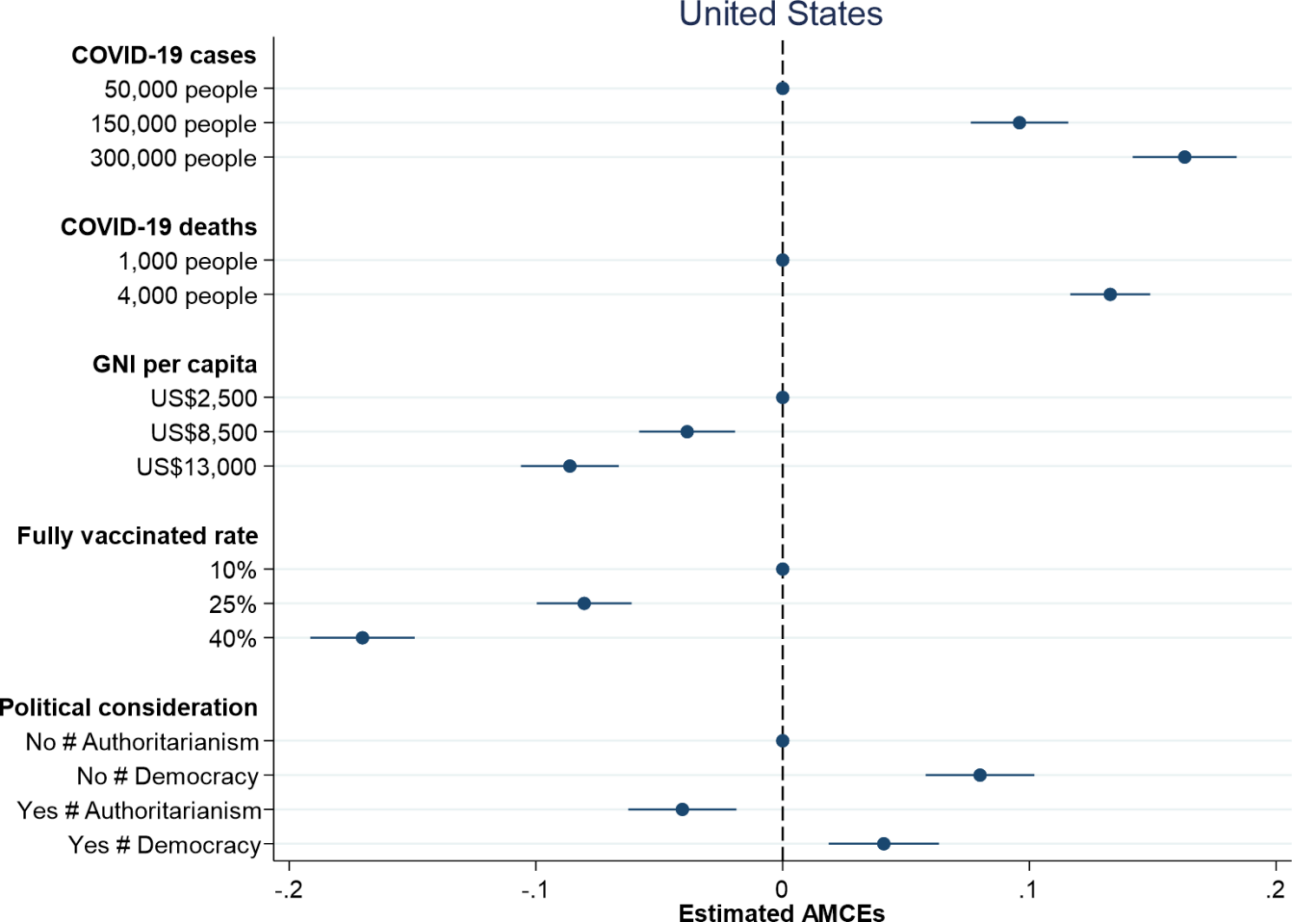
Probability of vaccine donation among American respondents by interacting formal diplomatic relation with political system

**Fig. 6:**
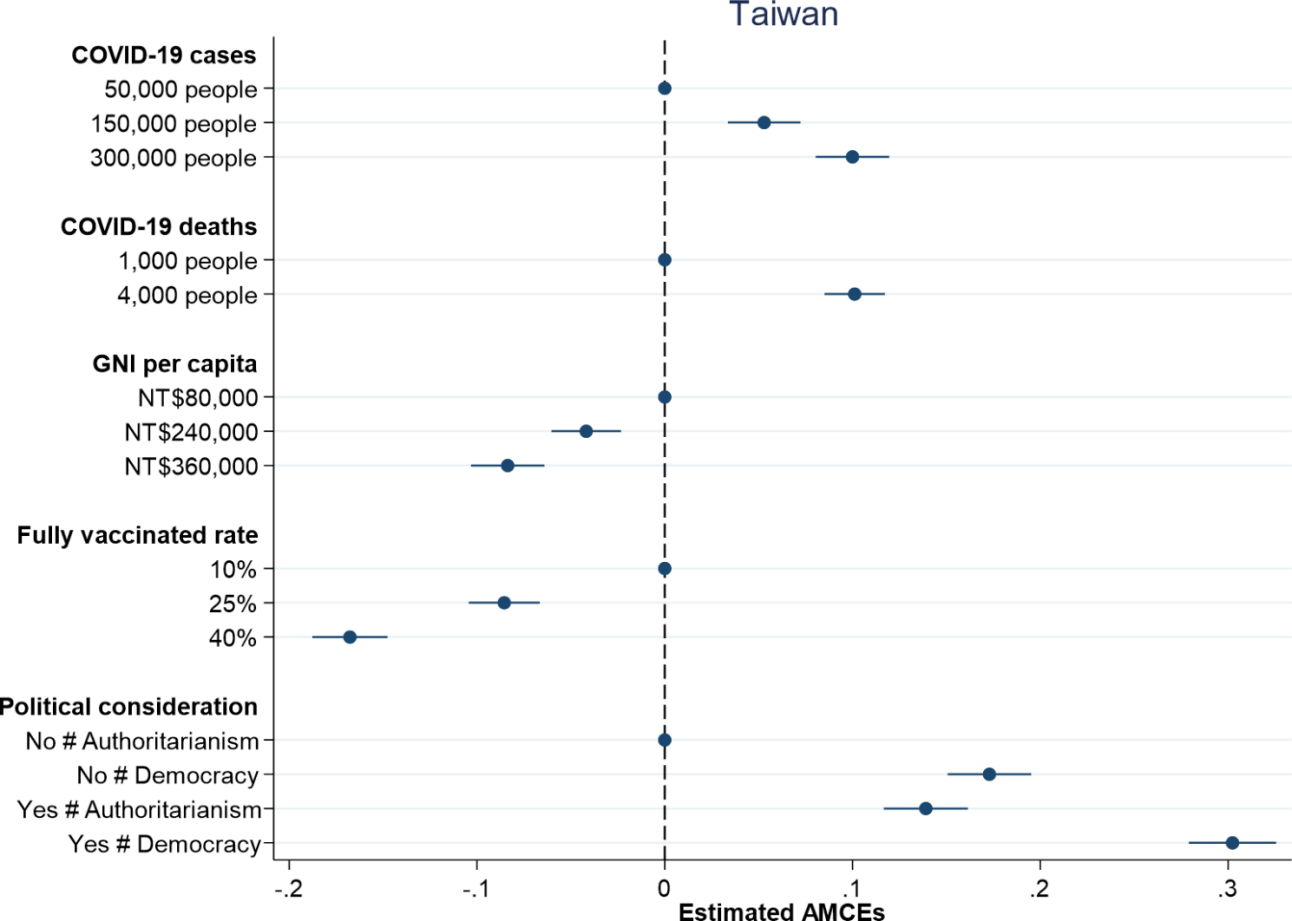
Probability of vaccine donation among Taiwanese respondents by interacting formal diplomatic relation with political system

**Fig. 7:**
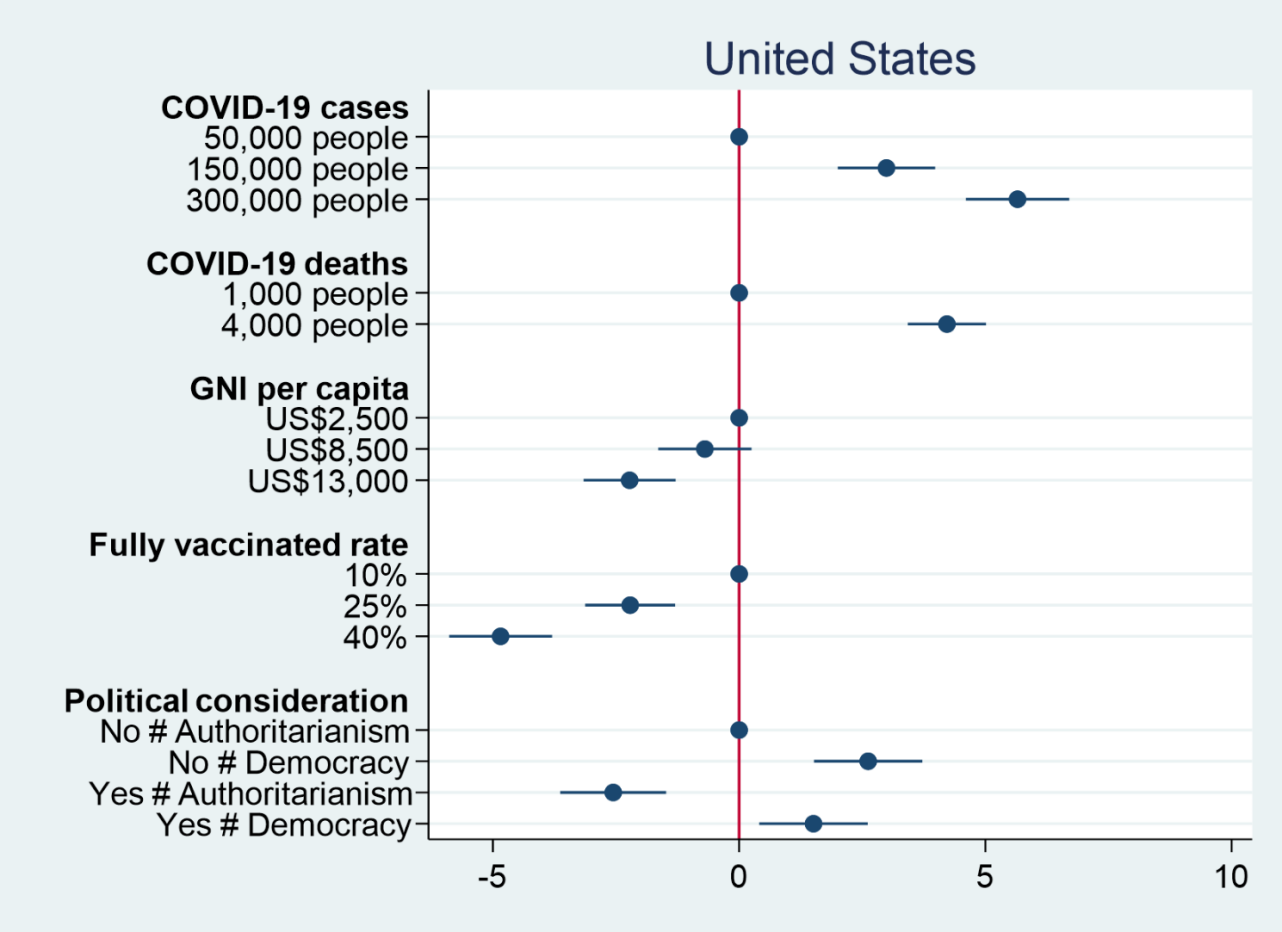
Percentage of vaccine donation among American respondents by interacting formal diplomatic relation with political system

**Fig. 8:**
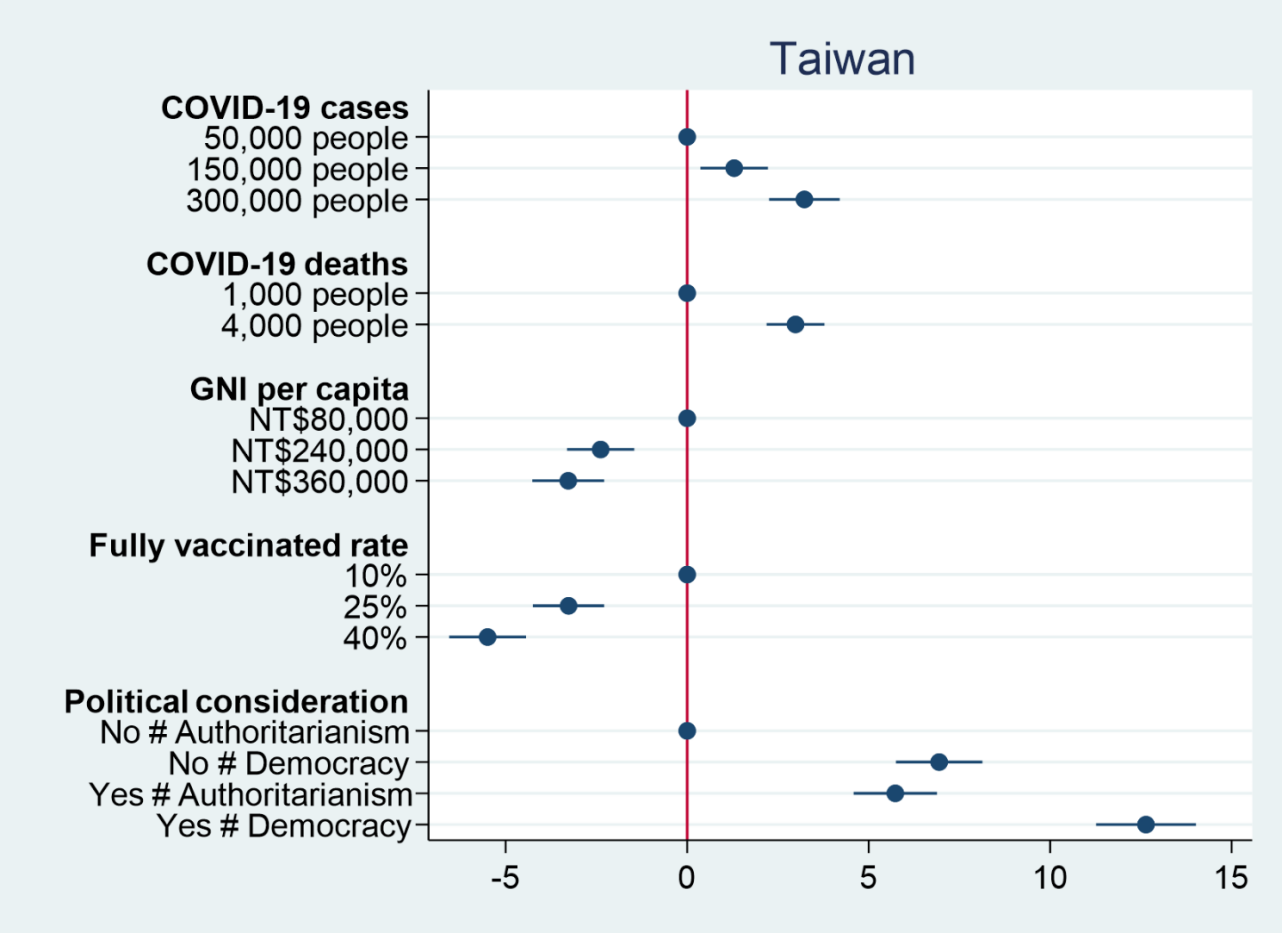
Percentage of vaccine donation among Taiwanese respondents by interacting formal diplomatic relation with political system

In the light of the heterogeneous effect of formal diplomatic relation, we further investigate whether people would view democratic and authoritarian countries differently depending on formal diplomatic relations. Thus, we use the attributes of formal diplomatic relation and political system to classify countries into four categories: authoritarian country with and without formal diplomatic relations and democracy with and without formal diplomatic relations. We perform conjoint analyses by regressing our two outcome variables on this new variable, namely political consideration, as well as the same variables related to damages due to COVID-19 and countries’ competencies. Since the effects of the variables gauging damages due to COVID-19 and countries’ competencies are consistent with those of our previous analyses, we focus our discussion on political consideration. As demonstrated in Figs. 5 and 6, both American and Taiwanese respondents prefer to donate vaccines to democracies regardless of whether there are formal diplomatic relations compared to authoritarian countries. More interestingly, we find that Taiwanese respondents prefer authoritarian countries having formal diplomatic relations with Taiwan to authoritarian countries without diplomatic relations in terms of vaccine donation. This implies that formal diplomatic relation plays a significant role in driving Taiwanese people’s decisions on foreign interactions even involving global public health issues. By contrast, we find that American respondents do not hold favorable attitudes toward authoritarian countries having formal diplomatic relations with the United States compared to authoritarian countries without diplomatic relations when making decisions on which country to donate vaccines. One possible explanation is that American people might think that authoritarian countries having formal diplomatic relations with the United States could receive assistance from the United States and thus they would rather give COVID-19 vaccines to those without diplomatic relations. In addition, when we turn to the percentage of vaccine donation, we find similar results as displayed in Figs. 7 and 8. While both American and Taiwanese respondents tend to donate more vaccines to democracies than authoritarian countries regardless of whether there are formal diplomatic relations, they have different preferences between authoritarian countries with and without formal diplomatic relations in terms of the percentage of vaccine donation. The results again confirm that American and Taiwanese respondents weigh differently formal diplomatic relation when deciding how many vaccines should be given to authoritarian countries affected by COVID-19.

## Discussion

The COVID-19 pandemic has thoroughly changed the life of people around the world. Given constantly emerging variants of COVID-19, we could only count on vaccines to protect ourselves. However, not all countries could get timely and equitable access to COVID-19 vaccines and that is why vaccine equity has been a major issue during the COVID-19 pandemic. Although developed countries have donated over half a billion COVID-19 vaccines to those countries in need, there has been still a huge variation in vaccination rates among countries. In view of the principle of vaccine equity, donor countries should apply the criterion of needs to make decisions about vaccine donation instead of considering recipient countries’ economic status. This study examines whether people follow the same criterion or take other factors into consideration to decide which country to donate vaccines and how many vaccines should be delivered. We conduct the conjoint experiment in the United States and Taiwan which are very different from each other in terms of culture and international status. While the results from both countries are mostly consistent, we also find some inconsistencies.

In line with the criterion of needs, both American and Taiwanese people prefer to donate vaccines and deliver more vaccines to countries with the high number of COVID-19 cases and deaths. However, contrary to the principle of vaccine equity, both American and Taiwanese people still consider recipient countries’ economic status to make decisions about vaccine donation. They are more willing to donate vaccines and deliver more vaccines to countries with lower levels of economic development. Combined with the findings on the percentage of fully vaccinated people, we conclude that when a country has the capability to deal with COVID-19, people tend to think that there is no need to help it and would like to offer more assistance to those countries that could not manage COVID-19. Furthermore, politics plays a significant role in people’s decisions about vaccine donation. Both American and Taiwanese people prefer democracies over authoritarian countries to donate vaccines. Nonetheless, Taiwanese people tend to donate vaccines and deliver more vaccines to countries having formal diplomatic relations with Taiwan compared to those without formal diplomatic relations. By contrast, American people would rather donate vaccines and deliver more vaccines to countries without formal diplomatic relations with the United States. Although the principle of vaccine equity is ideal for allowing everyone in the world to have access to vaccines, people might have different considerations when making decisions about vaccine donation. While the humanitarian perspective could provide some explanatory power for individual preferences over vaccine donation, politics and recipient countries’ capabilities are also key determinants of individual decisions about vaccine donation. Ideally, political considerations should not come into play in terms of global public health issues, but our findings show that it is not the case when people think about vaccine donation. Although our results reveal that American people prefer to donate vaccines to countries having no diplomatic relations with the United States, most people in the world should have similar attitudes toward vaccine donation as demonstrated in our findings from Taiwan because it is nature to help friends rather than strangers or even opponents.

These findings could be considered in both science and policy, particularly in how developed countries can achieve global COVID-19 vaccine equity. As the next-generation vaccines have been developed to fight against multiple variants of COVID-19 [29], we need to think about the equitable distribution of new vaccines [30]. The United States has reached an agreement with Moderna to buy 66 million doses of the company’s next generation of COVID-19 vaccine [31], and it is anticipated that developed countries will be major buyers of next-generation vaccines. Consequently, if we aim to end the global public health crisis of COVID-19, it is required to stick to the principle of vaccine equity and vaccine donation should only be based on the needs without discrimination on any ground such as recipient countries’ socioeconomic and political status.

Lastly, the focus of this study is limited to the United States and Taiwan. However, the findings regarding vaccine donation from these two countries may be generalizable to other countries. It is essential to acknowledge that each country has its unique political, cultural, and socioeconomic factors that may influence individuals’ attitudes and behaviors regarding vaccine donation. Nevertheless, this study’s employment of a survey methodology and conjoint experiment design allows us to identify factors that are likely to be generalizable across various contexts. For instance, this study uncovers that politics plays a crucial role in vaccine donation decision-making, which could be relevant in countries where foreign relations and political regimes impact people’s decisions on vaccine donation. Moreover, this study highlights the significance of humanitarian and capability considerations in vaccine donation decisions, which may be applicable across diverse cultures and countries. Despite this, it is crucial to recognize that generalizing the findings across different countries necessitates further research that accounts for the specific cultural, political, and socioeconomic factors that shape individuals’ attitudes and behaviors in each context. Consequently, we urge more investigations into public opinion on vaccine donation in various cultural and political settings to gain a more profound understanding of the factors that influence people’s decisions to donate vaccines. On the other hand, although political leaders, rather than people, have the authority to decide how to donate or distribute vaccines, they must think about what people think since public support is the key to making them hold power in a democracy. If political leaders do not take what people think into consideration to make policy decisions about vaccine donation, they might be punished in the next election since people demonstrate exceptional skepticism of foreign aid [32]. Therefore, political leaders should be more cautious about vaccine donation.

## Conclusion

This study utilized two surveys with the conjoint experiment design in the United States and Taiwan to investigate the driving factors behind people’s decisions to donate COVID-19 vaccines. The results revealed that, apart from humanitarian and capability considerations, political factors also significantly impact individuals’ decisions regarding vaccine donations. Given that democratic leaders require public support to stay in office, it is crucial for them to consider their citizens’ preferences regarding vaccine donations while simultaneously achieving global equity in COVID-19 vaccine distribution. Undoubtedly, when faced with a global health crisis, it is critical for countries to transcend political differences and provide medical assistance to individuals impacted by pandemics, irrespective of their country of origin. Overall, this study can make significant contributions to our understanding of vaccine donations. On one hand, the results allow us to identify the factors that influence individual decisions on vaccine donation. By recognizing these factors, policymakers and researchers can devise strategies to overcome obstacles and enhance vaccine donation facilitators. On the other hand, the findings highlight the significance of foreign relations and political regimes in shaping people’s willingness to donate vaccines, which can assist in devising strategies to promote international cooperation in the realm of global health issues. Finally, this study has potential limitations. Although conjoint analysis is a useful research method for examining individuals’ preferences and decision-making processes, this study simply focuses on how damage, national competency, and political consideration influence individuals’ decisions on vaccine donation, which may not completely reflect the real-world decision-making processes. People’s decisions regarding vaccine donation can be influenced by emotional and personal factors that may not be captured in this study. Furthermore, this study does not account for the complex social and cultural factors that may also influence individuals’ decisions to donate vaccines to other countries. Therefore, further investigation is required to examine the factors that influence individuals’ attitudes towards donating vaccines.

## Electronic supplementary material

Below is the link to the electronic supplementary material.

Supplementary Material 1

## Data Availability

The datasets used to generate Figs. 1, 2, 3, 4, 5, 6, 7 and 8 are available upon request.

## Abbreviations

AMCEs: Average marginal component effects
COVAX: COVID-19 Vaccines Global Access
COVID-19: Coronavirus disease 2019
GNI: Gross national income
WHO: World Health Organization
