# Supplementary material for: Politics matters for individual attitudes toward vaccine donation: cross-national evidence from the United States and Taiwan
Source: Global Health. 2023 Jun 20;19:40. doi: 10.1186/s12992-023-00940-x (PMC10283311; doi:10.1186/s12992-023-00940-x)
Supplement: Supplementary file 1 — Supplementary Material 1 [file 12992_2023_940_MOESM1_ESM.docx]

**Appendix. Experimental Design**

The respondents were presented with the following information before their participation in our online survey:

Welcome to join this study! You are going to participate in the survey conducted jointly by Greg Sheen (National Cheng Kung University), Yuan Hsiao (University of Washington), and Ching-Hsing Wang (National Cheng Kung University). We have to have your approval to ensure that you are willing to take part in this study. Please see the following information about this study.

1. This study aims at understanding individual political attitudes and how people understand issues related to the Covid-19 pandemic.
2. The respondents are people aged 18 or older in the United States.
3. This study will ask you some questions about your personal information, political attitudes and opinions about COVID-19.
4. There are no known risks to participate in this study.
5. Benefits: You can express your opinions. Besides, after completing this survey, you can earn points from Rakuten Insight and exchange the points you earn for popular rewards.
6. The questionnaire is answered anonymously and will not collect information that can be used to identify you. Therefore, your personal information will not be disclosed, shared or published. We will keep your personal information confidential, and research reports will only show the overall outcomes. When you participate in this study, you allow the researchers to use your information for research-related purposes. The research team will do its best to maintain your privacy and keep confidentiality to minimize possible risks.
7. The responses, in addition to confidential, will be treated solely for statistical and research purposes, and thus you will not be identified. Since this study will be published in academic journals, it will not derive commercial benefits.
8. Your participation is completely voluntary. During the survey, you can withdraw from the study without any reason.
9. The collected data will be stored and kept by the research team until December 2026 and will be released to researchers and students after the expiration date. The released data do not contain any personally identifiable information.
10. For any questions you can contact Greg Sheen, National Cheng Kung University, [gregcsheen@gs.ncuk.edu.tw](mailto:gregcsheen@gs.ncuk.edu.tw).

This study was approved by the Institutional Review Board (IRB) of National Cheng Kung University in Taiwan. If you have any questions about the rights of participating in the research or want to file a complaint, please contact the committee at: +886-6-2757575 ext. 51020, or email: [em51020@email.ncku.edu.tw](mailto:em51020@email.ncku.edu.tw).

Before you show your consent by clicking “agree” below, please read the form carefully to know more about the project.

Do you agree to participate in this research study?

□ Agree

□ Disagree

Only when the respondents clicked on the “Agree” checkbox can they proceed with the survey. Accordingly, we obtained voluntary and informed consent from the respondents. Besides, the following shows the instructions for the conjoint experiment:

*** Given the continual spread of COVID-19 around the world, many countries have an urgent need for vaccines. However, the global shortage and uneven distribution of vaccines have caused significant differences in the number of vaccines available across countries. In each of the following pages, you will be shown a table with two country profiles. For each pair, read each profile carefully and indicate how you want to distribute COVID-19 vaccines. ***

|  | **Country A** | **Country B** |
| --- | --- | --- |
| **COVID-19 cases** | This says the number of COVID-19 cases in the country. | |
| **COVID-19 deaths** | This says the number of COVID-19 deaths in the country. | |
| **Gross national income per capita** | This says the dollar value of a country’s final income divided by its population. | |
| **Fully vaccinated rate** | This says the percentage of fully vaccinated individuals in the country. | |
| **Formal diplomatic relation** | This says whether the country has formal diplomatic relation with the respondent’s country. | |
| **Political system** | This says whether the country is a democratic or authoritarian country. | |

Damage:

COVID-19 cases: 50,000 people, 150,000 people, 300,000 people

COVID-19 deaths: 1,000 people, 4,000 people

Need:

Gross national income per capita: US$2,500, US$8,500, US$13,000

Fully vaccinated rate: 10%, 25%, 40%

Political factor:

Formal diplomatic relation: Yes, No

Political system: democracy, authoritarianism

【The above characteristics are presented in a random manner and each respondent was asked to answer 5 times.】

1. If you have 1 million Covid-19 vaccine doses, which country would you give these vaccines to?

□ Country A □ Country B

1. If you could decide the percentages of distributing 1 million Covid-19 vaccine doses, what percentage of vaccines would you like to give the country you select in the previous question? Please note that the remaining COVID-19 vaccines would be given to the other country you do not select in the previous question. Please use the bar below to indicate the percentage of vaccines you want this country to receive.

0% 50% 100%

Country A/B
